# Airport Malaria in Non-Endemic Areas: New Insights into Mosquito Vectors, Case Management and Major Challenges

**DOI:** 10.3390/microorganisms9102160

**Published:** 2021-10-16

**Authors:** Leo Dilane Alenou, Josiane Etang

**Affiliations:** 1 Malaria Research Laboratory, Yaoundé Research Institute (IRY), Organization for the Coordination of Endemic Diseases’ Control in Central Africa (OCEAC), Yaoundé P.O. Box 288, Cameroon; leodilanealenou@gmail.com; 2 Department of Biological Sciences, Faculty of Medicine and Pharmaceutical Sciences, University of Douala, Douala P.O. Box 2701, Cameroon; 3 Department of Insect Biotechnology in Plant Protection, Institute for Insect Biotechnology, Faculty 09—Agricultural Sciences, Nutritional Sciences and Environmental Management, Justus-Liebig-University Gießen, Winchester Str. 2, 35394 Giessen, Germany

**Keywords:** air transportation, malaria, *Anopheles* mosquitoes, *Plasmodium* spp., non-endemic areas

## Abstract

Despite the implementation of preventive measures in airports and aircrafts, the risk of importing *Plasmodium* spp. infected mosquitoes is still present in malaria-free countries. Evidence suggests that mosquitoes have found a new alliance with the globalization of trade and climate change, leading to an upsurge of malaria parasite transmission around airports. The resulting locally acquired form of malaria is called Airport malaria. However, piecemeal information is available, regarding its epidemiological and entomological patterns, as well as the challenges in the diagnosis, treatment, and prevention. Understanding these issues is a critical step towards a better implementation of control strategies. To cross reference this information, we conducted a systematic review on 135 research articles published between 1969 (when the first cases of malaria in airports were reported) and 2020 (i.e., 51 years later). It appears that the risk of malaria transmission by local mosquito vectors in so called malaria-free countries is not zero; this risk is more likely to be fostered by infected vectors coming from endemic countries by air or by sea. Furthermore, there is ample evidence that airport malaria is increasing in these countries. From 2010 to 2020, the number of cases in Europe was 7.4 times higher than that recorded during the 2000–2009 decade. This increase may be associated with climate change, increased international trade, the decline of aircraft disinsection, as well as delays in case diagnosis and treatment. More critically, current interventions are weakened by biological and operational challenges, such as drug resistance in malaria parasites and vector resistance to insecticides, and logistic constraints. Therefore, there is a need to strengthen malaria prevention and treatment for people at risk of airport malaria, and implement a rigorous routine entomological and epidemiological surveillance in and around airports.

## 1. Introduction

Malaria is a parasitic disease transmitted by the *Anopheles* mosquito species, to which 3.5 billion people are exposed worldwide [1]. In 2019, 229 million new cases were recorded and the disease is endemic in 87 countries [1]. Although it has been eliminated in the United States, Canada, and Europe, more and more cases are reported in these regions, mostly imported cases [2]. Overall, it is estimated that over 30,000 annual cases of imported malaria occur among the travelers from endemic countries [3]. The majority of these travelers arrive from sub-Saharan Africa (97.5%), mainly the native Africans residing in Europe (82.2%) [4,5]. Indeed, malaria is a real emergency in these areas because it can rapidly become fatal if not diagnosed and treated promptly.

The epidemiological patterns of imported malaria are closely related to the travels of *Anopheles* mosquito vectors and humans infected with *Plasmodium* spp. parasites [6]. When local environmental conditions at the destinations are favorable for the development of *Anopheles* species (i.e., 27 ± 2 °C temperature and 70 ± 5% relative humidity), those which are efficient malaria vectors and capable of breeding near airports can cause local transmission of *Plasmodium* spp. parasites after blood feeding on infected travelers [5]. In addition, malaria parasite transmission in the vicinity of airports can result from infected *Anopheles* mosquitoes brought by aircraft, that can survive long haul flights and adapt for enough time in the new environment after arrival [7]. The resulting autochthonous (locally-acquired) malaria, in which cases are principally clustered around international airports, is known as “airport malaria” [8]. The patients had neither recently traveled to endemic areas nor recently received blood transfusions [9]. This form of malaria is increasingly being reported in Europe and North American countries that are considered malaria-free [9,10].

During the early 1930s, the World Health Organization (WHO) was already thinking about how to disinsectize airplanes in order to prevent airport malaria. Currently, the method recommended by WHO is disinsection by spraying a pyrethroid insecticide in the cabins and holds of aircraft flying from endemic areas [11,12]. However, mosquitoes in passenger luggage and cargo can escape this insecticide and survive during the flight, more importantly if they have developed resistance to pyrethroid insecticides [13]. Despite the implementation of prevention measures, the risk of importing infected mosquito vectors has increased with the intensification of air transport, as well as the size and complexity of today’s aircraft, with a multitude of niches that escape insecticide treatment. However, little research has been conducted to better understand the transmission of malaria at airports and around. Furthermore, piecemeal information is available, regarding the evolution of epidemiological patterns, as well as the challenges in its diagnosis, treatment, and prevention. Understanding these issues is a critical step towards a better implementation of control strategies. The purpose of this study is to draw the attention of researchers and policy makers to the concerns about airport malaria in non-endemic areas.

In this paper, we provide new insights into the factors that might lead to the re-emergence of airport malaria and potential actions to be taken in order to strengthen the strategies for preventing the reintroduction of malaria in malaria-free regions.

## 2. Data Retrieval

We conducted a systematic review of the literature published from 1969 (when the first cases of airport malaria were reported) to 2020 (51 years later), in four main databases, including Hinari Health, PubMed, Google and Google Scholar. Priority was granted to documents from the “Journal Article” category and other papers highlighting at least one of the following terms: “airport”, “malaria”, “prevention”, “*Plasmodium* spp.”, “*Anopheles*” “mosquitoes”, “non-endemic areas” in their titles, abstracts, or keywords.

Advanced searches conducted in databases were based on search equations, which represent associations of at least two of the selected terms. The research equations were formulated in English and French.

For the selected articles, full texts were obtained and examined in-depth to explore how airport malaria in non-endemic areas was documented. From each of the included articles in this review, information and data on the following topics related to airport malaria were extracted when available:

- Vector bionomics, incrimination, and importation from endemic areas;
- Airport malaria epidemiological profiles;
- Facilities and tools for diagnosis of *Plasmodium* spp. infections;
- Treatment and management of malaria cases;
- Airport malaria mass prevention strategies.

Epidemiological data were entered into a Microsoft Excel spreadsheet for easy access and data analysis. We also collected information on the study locations and grouped them into regions according to the United Nations designation of areas and regions [14]. Furthermore, we paid particular attention to the challenges encountered in the implementation of prevention and control strategies.

## 3. Results

**Figure 1:**
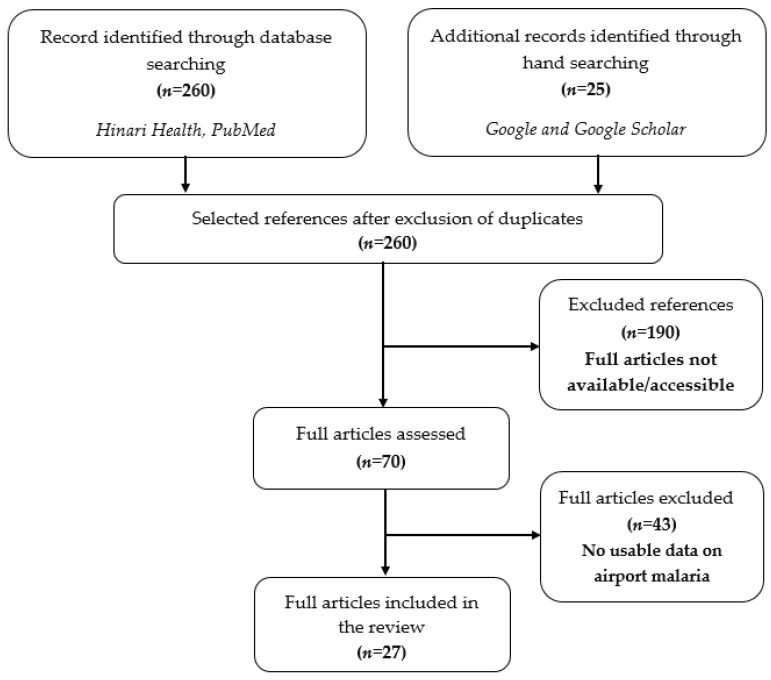
PRISMA chart showing the steps for the selection of articles.

The search resulted in 285 published articles from which more than 235 articles were excluded because they were not related to airport malaria or did not report data from non-endemic areas, as shown in Figure 1.

The 27 papers that were included in this work were those in which malaria cases in patients were associated with *Anopheles* transportation by aircraft or ships.

Of the 27 published papers, 52% (14/27) were specific to Europe, 37% (14/27) were multiregional, and 11% (3/27) for other countries (Americas, Tunisia, Turkmenistan). A total of 33 studies were recorded in the 27 selected articles. Among these studies, 33.3% (11/33) were about vector bionomics, incrimination, and importation from endemic areas, 21.2% (7/33) about malaria epidemiological profiles, 15.2% (5/33) about facilities and tools for diagnosis of *Plasmodium* spp. infections, 9.1% (3/33) about treatment and management of cases, and 21.2% (7/33) about mass prevention strategies, as shown in Table 1.

### 3.1. Mosquito Vectors

#### 3.1.1. Vector Bionomics and Incrimination

In Europe and the Mediterranean region, malaria elimination was achieved by the middle 20th century [41,42]. *Anopheles sacharovi*, *An. atroparvus*, *An. artemievi*, *An. beklemishevi*, *An. daciae*, *An. labranchiae*, *An. maculipennis*, *An. martinus*, *An. melanoon*, *An. messeae*, and *An. persiensis* were the main vectors of *Plasmodium* spp. parasites due to their anthropophilic behavior [17]. These 11 species are members of the *An. maculipennis* complex, and they are still widely distributed throughout the European Region [18]. Four of these 11 species, *An. sacharovi*, *An. atroparvus*, *An. labranchiae* and *An. messeae* are considered dominant vectors of malaria [19].

##### *Anopheles* *sacharovi*

*Anopheles sacharovi* has the most southerly distribution owing to its high adaptive capacity at adult and larval stages. It can breed in different types of water bodies, such as swamps, marshes, riverbanks, streams, ponds, and ditches. It is the most efficient mosquito vector of *Plasmodium* spp. parasites in southern Europe and the Middle East [18,19]. Currently, this species is involved in the transmission of *P. vivax* malaria in Iran [20,21], Iraq [43], and Turkey [44]. It is also responsible for the reemergence of malaria in Georgia, Armenia, and Azerbaijan [18]. Female mosquitoes have opportunistic blood feeding behavior and can feed on any available host, including man, cow, sheep, chicken, horse, and donkey [18]. *Anopheles sacharovi* has been found resistant to DDT [21] and dieldrin [45]. Such ecological and behavioral plasticity, together with emerging insecticide resistance, has prevented the elimination of *An. sacharovi* in Israel, Greece, and Turkey [18,44].

##### *Anopheles* *atroparvus*

*Anopheles atroparvus* is present in Eastern and Central Europe, as well as in the United Kingdom [22,46]. Its absence has been reported in Greece, Turkey [47], and southern Italy [22]. Although described as a species with a preference for brackish larval habitats [23], this species has been found in a number of freshwater habitats, including canals, ditches, riverbanks, pools in riverbeds, and rice paddies [19]. *Anopheles atroparvus* has been described as an endophilic species, mostly endophagous and zoophilic [19]. Its hosts, in order of preference, are the rabbit, horse, cow, pig, and sheep [48,49,50,51]. Because of its association with human settlements, *An. atroparvus* also exhibits anthropophilic behavior [19]. Historically, *An. atroparvus* was involved in the transmission of local strains of *P. vivax* [52] and *P. falciparum* in Spain [22]. Studies have shown that this vector species is refractory to Asian and African *P. falciparum* [19,22]. De Zulueta et al. [53] and Cambournac [54] though that the refractory character of *An. atroparvus* with African and eastern *P. falciparum* strains is a “fact undeniable”. However, Sousa [55], under laboratory conditions, succeeded in infecting *An. atroparvus* with tropical strains of *P. falciparum*. Thus, *An. atroparvus* can be infected with tropical strains of *P. falciparum*, although its transmission would unlikely occur under natural conditions.

##### *Anopheles* *labranchiae*

*Anopheles labranchiae* is widely present in central Italy despite the eradication campaign launched in 1946 [56]. In general, its larvae are found in stagnant or low flowing water [57] and rice fields [58,59]. Females can aggressively attack human hosts and are de-scribed as “persistent” in their attempt to enter rooms during the night [59]. Although de-scribed as zoophilic, this species is opportunistic in its choice of host; it can alternatively bite humans or animals [58,60,61]. As with *An. atroparvus*, some studies have shown that *An. labranchiae* is refractory to tropical strains of *P. falciparum* [53]. However, Toty et al. [62] reported historical evidence, as well as the results of a contemporary study conducted by the Centre de Production et d’Infection d’Anophèles (CEPIA) in Paris where 14% (13/99) of specimens of *An. labranchiae* were experimentally infected with the laboratory-grown African strain of *P. falciparum*. This study also detected sporozoites in the salivary glands of three specimens, indicating that *An. labranchiae* is not only susceptible but also potentially capable of transmitting at least some strains of African *P. falciparum*. 

##### *Anopheles* *messeae*

*Anopheles messeae* is the most widespread species of the *An. maculipennis* complex [23]. Its distribution extends from Europe to Asia passing through Russia [63]. Di Luca et al. [59] identified a number of genetic polymorphisms within *An. messeae* and defined five distinct haplotypes associated with different geographical areas. However, they could not confirm whether this polymorphism was associated with behavioral variability at different places. In surveys conducted in Slovenia and Croatia [64,65], the authors found adult *An. messeae* larvae in swamps, stagnant freshwater marshes, alluvial plains, and at sites near large lakes. Takken et al. [66] also inferred the presence of *An. messeae* in more brackish habitats. In urban areas, females have been found inside human dwellings [67]. Considering its high degree of zoophily and outdoor biting behavior, some authors thought it is very unlikely that this mosquito species is involved in local malaria transmission [66,68]. In contrast, Fyodorova et al. [67] found that 40% of female *An. messeae* collected in urban areas contained human blood, with the remaining 60% having fed on cats (40%) and chickens (20%). Becker et al. [69] stated that “blood meals are taken from humans only when the density of *An. messeae* is very high and there is a shortage of livestock”. Some evidence suggests that *An. messeae* may be refractory (or essentially refractory) to tropical strains of *P. falciparum* [70]. Indeed, the status of *An. messeae* as a malaria vector has been questioned, especially since the discovery of a new species in 2004, named *An. daciae*, widely distributed in Romania, southwest England, and parts of southern and central Finland [19,71]. *Anopheles daciae* can only be distinguished from *An. messeae* using egg morphology or by sequencing the internal transcribed spacer 2 (ITS2) of ribosomal DNA and the cytochrome oxidase 1 (COI) of mitochondrial DNA [19]. It is therefore possible that *An. daciae*, and not *An. messeae* is involved in malaria transmission.

#### 3.1.2. Suspected Vectors

In addition to proven malaria vectors, there are also other vectors of the *Hyrcanus* group that may play distinct roles in the transmission of malaria pathogens, in particular *P. vivax* [72]. The *Hyrcanus* group includes approximately 30 closely-related species of mosquitoes distributed in the Palearctic and Eastern regions [73]. Among these 30 species, *An. sinensis* and *An. lesteri* are the main vectors of malaria in China [74]. *Anopheles hyrcanus* s.s. is a potential malaria vector in southern France and central Europe [48]. *Anopheles kleini* and *An. pullus* are the main vectors of malaria in the Republic of Korea [75], while *An. sinensis*, *An. nigerrimus* and *An. peditaeniatus* are potential malaria vectors in Thailand [76,77]. Within this group, there is some confusion as to the taxonomic status of *An. hyrcanus* s.s. and *An. pseudopictus* [15,16]. They were previously considered synonymous species. However, recent studies and identification keys refer to their taxa either as distinct species or as synonymous species. According to Miaoulis et al. [16], there is no strong evidence for treating these taxa as different species, so they concluded that *An. hyrcanus* s.s. and *An. pseudopictus* should be reclassified to their former synonym status.

#### 3.1.3. Vector Importation

In 1930, shortly after the intensification of steamship traffic between the African and South American continents, *An. arabiensis*, a major malaria vector in Africa, was observed in the state of Natal in Brazil [24]. In 1994, 2000–5000 *Anopheles* mosquitoes were estimated to be imported in France within a week, i.e., 8–20 *Anopheles* specimens per flight [9]. In 2013, the presence of an Asian anopheline species (*An. stephensi*) was discovered in urban areas to Djibouti, and in subsequent years in Ethiopia [78]. More recently, in 2017 the mosquito species *An. bancroftii* was reported to be introduced in New Caledonia in an area nearby the airport of Noumea. New Caledonia is a French island in the southern Pacific Ocean which had been free of *Anopheles* mosquito species vectors of human *Plasmodium* spp., making this island free of malaria transmission [79,80]. The investigations suggest that the importation of this mosquito species was made by aircraft, although the route of this introduction was not clearly identified [81].

Since 1969, locally transmitted malaria cases (acquired on the European continent) have been associated with infected anophelines from endemic areas. Germany, the Netherlands, Spain, France, Italy, Greece, and Turkey have reported large numbers of cases [13]. Studies indicate that during the summer months (May-September), it is likely that a tropical mosquito such as *An. gambiae* can successfully establish itself in temperate regions of Europe or North America [7,25].

### 3.2. Airport Malaria Epidemiological Profiles

**Figure 2:**
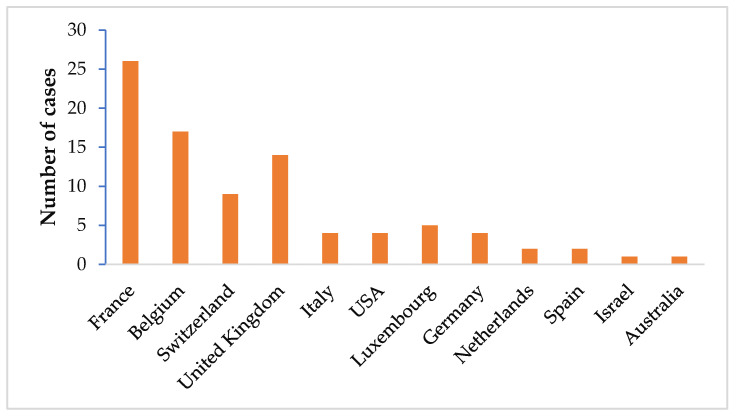
Countries in which confirmed or probable cases of airport malaria have been reported in 2006 [6].

The global epidemiological data reported in 2006 showed that countries mostly affected by airport malaria are in Europe, particularly France (29.2%), Belgium (19.1%) and Switzerland (10.1%), Luxembourg (5.6%), Italy and Germany (4.5%), followed by the UK (15.7%), USA (4.5%), Israel and Australia (1.1%) (Figure 2) [6]. Some cases have also been reported in Tunisia [82]. In the meantime, this epidemiological situation may be changed, with the intensification of international trade.

In France in 2008, a man and his wife living in Paris and who never travelled to a malaria endemic area, were hospitalized at the University Hospital Central (CHU) of Nice for uncomplicated *P. falciparum* malaria. These people were living in Saint Mard (a town located 7 km from Roissy Charles de Gaulle Airport) [33]. In 2013, four cases of airport malaria due to *P. falciparum* were notified for the first time in Tunisia; all these patients were neighbors living within 2 km around the Tunis International Airport [82]. In Germany in 2019, two employees of Frankfurt International Airport, working in aircraft maintenance were hospitalized with severe *P. falciparum* malaria at the University Hospital Frankfurt [31]. The airplanes under maintenance were returning from three *P. falciparum* malaria endemic countries in sub-Saharan Africa. Similar cases of airport malaria due to *P. falciparum* have been reported in other countries. Furthermore, there has been an increasing number of human *P. knowlesi* malaria cases imported from Southeast Asia to other areas across the world [83]. *Plasmodium knowlesi* is a malaria parasite species commonly found in primates, which is now spreading in the human population in Asia. Indeed, numerous studies have shown an increase in the incidence of malaria due to this species among people returning from countries such as Indonesia, Singapore, the Philippines, Vietnam, Thailand, and Malaysia, where malaria has considerably increased during the last decade [84,85]. Over time, environmental changes, particularly those associated with extensive deforestation and global warming, may have resulted in an increase of the densities of zoophilic mosquitoes and their ability to feed on human hosts, thereby fostering the transmission of *P. knowlesi* to humans [83]. The rapid onset *P. knowlesi* parasite species in humans is therefore considered a leading factor for the upsurge of airport malaria.

**Figure 3:**
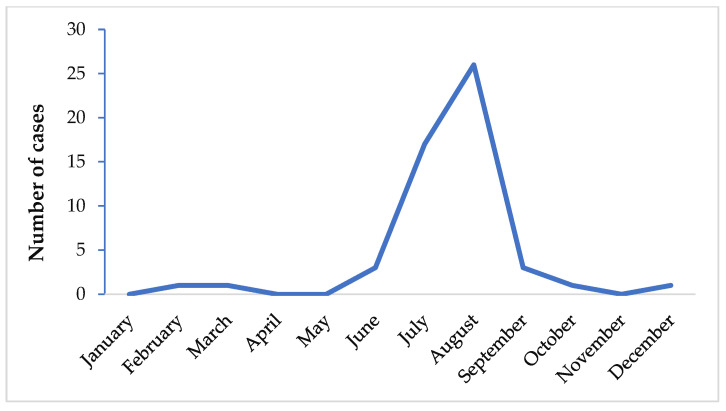
Airport malaria: period of the year in Europe when the greatest number of infections occurs [6,8,25,26,86,87,88].

It has been shown that the greatest number of airport malaria cases occurs in July and August (Figure 3) [6,25]. At that period of the year, the climate is similar to that of malaria endemic countries and therefore suitable for *Anopheles* spp. survival at their arrival, with subsequent development and transmission of *Plasmodium* spp. in non-endemic areas.

**Figure 4:**
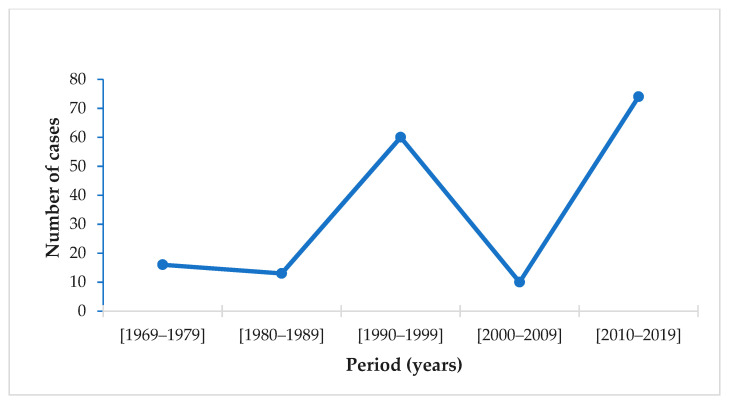
Airport malaria in the European Union: number of cases from 1969 to 2020 per 10-year period [9,25,26,28,86].

The evolution of the number of cases in Europe from 1969 to 2020 reveals an increase in the number of cases during the last decade (Figure 4). Among the 29 European Union countries reporting malaria data between 2010 and 2020, 11 reported airport malaria cases [25,28]. According to WHO, airport malaria cases have been on the rise since 2015 [42]. In France, mortality due to airport malaria is 20 times higher than that of imported malaria [24].

### 3.3. Facilities and Tools for Diagnosis of Plasmodium spp. Infections

**Figure 5:**
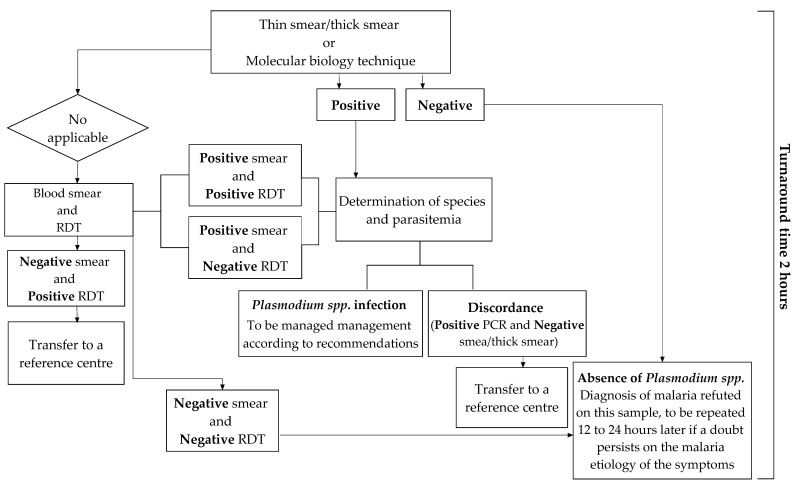
Flow chart showing the process of biological diagnosis of imported malaria [35].

The diagnostic tools that can be used in the laboratory differ in their technologies, sensitivities, interpretations, and costs. The use of either can be prioritized according to the technical resources available and the biologist’s experience in detecting *Plasmodium* spp. parasites, as shown Figure 5 [35].

The 12th consensus conference on clinical practice organized by the Society of Infectious Pathology of French Language (SIPFL) in 1999 and revised in 2008 recalled that the biological diagnosis of malaria should be microscopic [89]. It is the method that objectively determines the presence of the parasites. This examination must allow to obtain results in less than two hours. However, in airport or imported malaria, low parasitemia often leads to performing a concentration technique (thick drop) or QBC™ Malaria Test as the first line [33]. The QBC™ Malaria Test is the fastest and most sensitive technique. Its extra cost compared to the thick drop is largely balanced with time savings. Still, the blood smear remains essential to determine *Plasmodium* spp. [24]. These techniques must therefore be perfectly known and mastered by the medical professionals in place; therefore, regular hands-on training is essential for them. However, evaluations conducted in France have shown that nearly 30% of laboratories returned their diagnosis results in more than two hours [33]; furthermore 16.5% of the results were inaccurate [90]. To overcome these challenges, there is a renewed interest in non-microscopic techniques.

Rapid Diagnostic Tests are the most accessible and least expensive; their ease of use makes them of valuable aid [33]. They are highly used by laboratories that rarely see positive blood samples and by biologists or technicians on-call [91]. It should be recalled, however, that this test is less sensitive (e.g., 10 fold less) than the concentration methods; so, its negativity does not make it possible to rule out the diagnosis in the case of very low parasitemia [33]. This is true for *P. falciparum* and even more so for other species [34,92].

Molecular biology techniques offer the advantage of a very high sensitivity. Most of the time, they are used as confirmatory tests in cases where the interpretation of the thick drop is difficult [33,34]. Venous blood (300 µL) is applied to a filtered paper FTA (Flinders Technology Associates) microcard, which is then stored in an aluminum barrier bag until the PCR (Polymerase Chain Reaction) test is performed [93]. The structure and composition of the FTA blotting paper allows for automatic isolation of DNA from its surface; the DNA is ready to be purified and analyzed after 30 min [93]. However, due to their implementation constraints, these techniques can only be used in equipped centers and during regular working hours. Indeed, they may not be available on call; therefore, their utilization is often delayed.

The diagnosis of *Plasmodium* spp. in mosquitoes can be determined by three main methods:

- Microscopically examining the salivary glands of mosquitoes after dissection for direct observation of *Plasmodium* spp. sporozoites;
- The circum-sporozoite antigen enzyme-linked immunosorbent assay (CSP-ELISA);
- Analysis of genomic markers using polymerase chain reaction (PCR).

These methods allow the determination of the sporozoite index (SPI) of *Anopheles* and the entomological inoculation rate (EIR) [94], which is the number of infective mosquito bites per human per night. Although the first two methods can routinely be used in specialized laboratories of entomology, they are known to be labour intensive and it has been shown that CSP-ELISA [95] overestimates the real infection rate by detecting the CSP from the oocysts bursting 2–3 days before the sporozoites actually reach the salivary glands [96,97]. Research efforts in recent decades have led to the development of molecular biology tools for detecting the five major species of *Plasmodium* (*P. falciparum*, *P. malariae*, *P. ovale, P. vivax* and *P. knowlesi*) in human blood [98,99] and in *Anopheles* mosquito samples [100]. In the multiplex PCR assays, a Small Subunit of ribosomal RNA (SSU rRNA) of each *Plasmodium* spp. can be detected, but it requires a significant amount of parasite DNA, which is not easily achieved with small tissues such as a single pair of salivary glands.

Now, specific and sensitive methods such as quantitative PCR (qPCR) have been developed to measure the prevalence and intensity of *Plasmodium* spp. infections in human blood samples [101,102], as well as in mosquito samples [103,104].

### 3.4. Treatment and Management of Malaria Cases

Following the expansion of malaria cases in the European Union, the pharmaceutical policy for the treatment of malaria has gradually changed over the years from chloroquine and mefloquine monotherapies used as first-line treatment for uncomplicated malaria to artemisinin combination therapies (ACT) [35,37,105].

For adult treatment, four antimalarial drugs from three different therapeutic classes are routinely recommended [36,106]; these include Artenimol—piperaquine, Artemether—lumefantrine, and Atovaquone—proguanil; the advantages and disadvantages of each of these drugs are summarized in Table 2.

For pregnant women, because of the embryotoxicity and teratogenicity of artemisinin, ACTs are not recommended in the first trimester of pregnancy. The treatment of uncomplicated *P. falciparum* malaria is based on quinine or, failing that, atovaquone—proguanil during the first trimester. From the second trimester onwards, artemether—lumefantrine is the preferred treatment [38]. For children, the first-line treatments are artemether—lumefantrine; artenimol—piperaquine as shown in Table 3. Mefloquine and atovaquone—proguanil are second-line drugs used in cases of intolerance, contraindication, or failure of ACT treatment. Oral quinine is the third-line drug [35,38].

The choice of these treatments is based on precise clinical and biological criteria, the existence of possible contraindications, the frequency and importance of side effects (particularly those that are potentially severe), the way the drug is taken, and its cost [35,107]. In young children and others at risk of complications (advanced age, co-morbidities, pregnancy, splenectomy), initial hospitalization in an intensive care unit is recommended for the first 48 h, even in the absence of severe criteria [35]. After a minimum incubation period of at least six days, *P. falciparum* malaria manifests itself mainly as a fever [108]. However, it may be absent at the time of consultation. The associated clinical signs are not specific but headaches are common. Digestive symptoms (nausea, diarrhea, transit disorders) and cough are often present, whatever the age [31]. Therefore, it is essential to look for signs of severity and bacterial co-infection. During management, clinical examination is repeated to detect symptoms suggestive of an early severe form (somnolence, even minimal vigilance disorders, conjunctival subicterus, haemodynamic disorders, dyspnoea) [107]. However, even in the absence of severity criteria, frail patients are treated with intravenous quinine [105].

### 3.5. Airport Malaria Mass Prevention Strategies

Malaria prevention at airports relies heavily on vector control with an insecticide [39]. International health regulations recommend that airports and 400 m perimeter surrounding areas be disinsected to make them free of *Anopheles* vectors of malaria [13]. In addition, all aircraft and ships (civil, military, or private) arriving from areas at risk of mosquito-borne diseases, particularly from Africa, Asia, the Middle East, and the Indian Ocean islands, must be disinsected before landing [39]. The current recommended dose of insecticide for aircraft spraying is 2% permethrin (25/75), i.e., 0.7 g active ingredient per 100 m^3^ in various parts of the aircraft, including the cockpit, passenger cabin, cargo compartments, toilets, and overhead/side garment compartments [39,40]. Pre-flight spraying is carried out by crew members just before departure [40]; this measure is under the responsibility of the airlines.

### 3.6. Major Challenges

Currently, all actions undertaken in the fight against malaria at airports encounter biological and/or operational challenges; below are some of the key challenges.

#### 3.6.1. Which Case Definition Should Be Considered?

Annual reporting of suspected malaria cases by the European Centre for Disease Prevention and Control (ECDC) really started in 2007 [25], following an upsurge of malaria and infectious diseases in the European Union (EU). In 2019, 29 EU and European Economic Area (EEA) countries reported malaria data [25]; among these countries, 27 reported case-based data and 2 reported aggregate data (Belgium and Bulgaria). Twenty-six countries used the EU case definition, two (France and Germany) used another case definition, and one (Belgium) did not specify the case definition used [25,26]. Malaria surveillance is comprehensive but essentially passive. Reporting is mandatory in 26 countries, voluntary in France and Belgium, and classified among the “other” diseases in the UK [25].

In Canada, malaria has been a nationally notifiable disease since 1983 [109]. However, only laboratory-confirmed cases with evidence of *Plasmodium* spp. in a blood smear are reported to the federal authorities. Reported malaria cases are classified into five categories [109], as follows:

- Induced cases, i.e., confirmed cases of malaria contracted through a blood transfusion from a donor with confirmed parasitemia;
- Indigenous cases, i.e., confirmed case of malaria contracted through a mosquito bite in Canada;
- Imported cases, i.e., confirmed case of malaria contracted outside Canada;
- Confirmed congenital cases i.e., confirmed case of malaria in an infant up to 3 months of age who has not left Canada since birth and for whom the parasite has been confirmed in the mother;
- Probable congenital cases, i.e., confirmed case of malaria in an infant up to 3 months of age who has not left Canada since birth, but for whom the parasite has not been confirmed in the mother. Cases of probable malaria are reported for public health management purposes only.

This classification differs from the WHO classification. In areas where laboratory diagnostic services are available, WHO classifies malaria cases as follows: asymptomatic malaria, confirmed uncomplicated malaria, confirmed severe malaria, and confirmed death from malaria [110].

#### 3.6.2. Delay in Diagnosis and Treatment

In areas of moderate to intense transmission of *Plasmodium* spp. parasites, people may develop a form of immunity (premonition) after several years of regular exposure to infected mosquito bites. This is a state of protection against the disease, but not against the parasite. Parasitemia usually remains very low and the patient does not develop severe forms of the disease.

In non-endemic regions, the entire population is considered systematically as non-immune. The particular severity of malaria in these areas is due to delays in diagnosis and treatment, as well as a lack of malaria immunity in infected individuals [109]. Of the cases reported to the Canadian Malaria Network, only 20% visited a health care facility within 24 h after symptoms onset and 44% waited more than three days [109,111]. Diagnosis by health professionals was delayed for more than 24 h in 34% of cases [111]. Thirty percent (30%) of patients developed the severe forms of the disease [109].

#### 3.6.3. Non-Compliance with Aircraft Disinsection Regulations

Until 1994, all flights from endemic areas were subject to health screening, according to WHO recommendations [112]. But due to the large number of flights today, controls have quickly become random [112]. After the surge of airport malaria cases in France in 1994, the local health authorities called attention of the airlines to the danger of importing mosquito vectors via flights from malaria endemic regions (sub-Saharan Africa, Southeast Asia, and South America) [13]. Subsequently, the frequencies of infringements in controlling at risk flights significantly decreased [13], e.g., from 26% in 1995 to 13% in 1996. The most common offenses were non-disinfection of the cargo hold (48%) and cabin (13%), insufficient use of aerosols (18%) and incomplete forms (12%) [13]. At the global level, WHO provided new international health regulations in 2005, which had a fairly high compliance rate (77%) [113]. Of the 23% non-compliance, 12% were due to poorly applied procedures or documentary traceability problems, and 11% concerned a lack of disinsection [113].

More critically, disinsection of incoming or outgoing aircrafts is not a requirement for some countries. Therefore, aircrafts registered in countries not covered by disinsection regulations, but flying to countries where this procedure is in force, are required to comply with the regulations.

#### 3.6.4. Drug and Insecticide Resistances

According to estimates, the global malaria incidence has declined by 27% between 2010 and 2015, but this downward trend slowed after 2015, falling by less than 2% between 2015 and 2019 [25]. Progress in the fight against malaria is still stagnating, especially in African countries where the malaria burden is very high [1]. Among the biological challenges reported are parasite resistance to drugs and vector resistance to insecticides.

At present, despite the wide range of antimalarial drugs available for the treatment of malaria, the treatment regimens put in place are all rapidly coming up against the development of drug resistance in *Plasmodium* spp. parasites [114]. Parasite resistance to certain antimalarial drugs, particularly those originating from the regions of Southeast Asia (Greater Mekong) or South America, where there is resistance to quinine, pyrimethamine-sulfadoxine, and artemisinin, is now spreading throughout the world [115,116]. Despite the emergence of resistance in endemic countries, ACTs remain the mainstay treatment for *P. falciparum* malaria in all EU/EEA countries [37,117]. However, there is very little data currently available on antimalarial drug resistance in these areas. Meanwhile, ACTs can also fail there due to both decreased sensitivity to artemisinin compounds and resistance to the associated drugs [115]. In France, ACTs are increasingly used routinely, delivered by hospitals and private sector pharmacies. This further increases the risk of inappropriate, incomplete, or incorrectly dosed treatment. Under these conditions, resistance to anti-malarial drugs could take hold more quickly.

In vector control, the prioritization of long-lasting insecticide-treated net (LLIN) distribution campaigns and indoor residual spraying (IRS) over the past decade has resulted in a 37% decrease in the number of new cases worldwide and a 65% decrease in the mortality rate among children under 5 years old [118,119]. However, the malaria burden in sub-Saharan Africa is still a great concern due to high disease transmission and the rapid increase of vector resistance to insecticides. In many areas, mosquitoes are resistant to permethrin, the pyrethroid insecticide used to disinfect aircrafts [120]. This resistance, which was first reported in Côte d’Ivoire in the major malaria vector in sub-Saharan Africa *An. gambiae*, is now widespread in Central, West, and East Africa [120,121,122]. In addition, although pyrethroids are less toxic to mammals than other insecticides, people who are temporarily or regularly exposed to them could be exposed to health risks [123]. Therefore, there is a need for alternative vector control measures in and around airports, e.g., non-chemical interventions to mitigate vector resistance to insecticides.

## 4. Discussion

From this review, it appears that the risk of malaria transmission by mosquito vectors breeding in non-endemic countries is not zero. Moreover, this risk is more likely associated with infected tropical vectors coming in by air or sea transportation. Indeed, there is ample evidence that airport malaria is re-emerging in non-endemic areas. The number of cases recorded during the last decade was 7.4 times higher than that observed during the 2000–2010 decade. This increase may be due to climate change, intensification of international trade, the decline of aircraft disinsection, in addition to delays in diagnosis and treatment.

Global warming has become an undeniable and tragic reality, more or less perceived by the world’s population as a whole, and whose scale and impacts on malaria transmission are increasing. Since the publication of the first report of the IPCC (Intergovernmental Panel on Climate Change) in 1990, there is a warning about the rise in temperature and its consequences [124]. Previous predictions on the increase of temperature are now becoming a reality. Whether humans will be able to stop this inexorable increase, for which there is no other option than drastically reducing our greenhouse gas emissions, is questionable. According to the French public meteorological institute, the 2010–2020 decade was the hottest period from least since 1980 [125,126]. This has resulted in an increase in the number of days with average temperatures exceeding 25 °C in summer [125]. On the other hand, the cases of airport malaria observed in the last decade occurred mainly in summer between June and September, when climatic conditions were favorable to the survival of the mosquitos (average temperature 23 °C, range: 17–31 °C) [127]. One of the key factors controlling mosquito development both in water and on land, as well as parasite development in mosquitoes, is the temperature. Indeed, the duration of sporogony, mosquito longevity, and the duration of larval development are closely influenced by the temperature [128].

On a global scale, human populations passed the 7 billion mark in 2011 [129]. This has resulted in an increase in the number of international trips and airlines have increased the number of flights across the world’s capitals. It should also be noted that most of the patients suffering with airport malaria live near or worked at an international airport. Field studies revealed that these patients are infected by vectors coming inland by air transportation, i.e., which escaped the chemical treatment of aircrafts. Nowadays, aircraft disinsection is declining in most countries. Although WHO continues to recommend disinsection of aircrafts, there are concerns about the health effects of pyrethroid insecticides used and the resulting lawsuits have led many airlines and governments to stop the spraying practices [6]. To strengthen the capacity of airlines, the ICAO (International Civil Aviation Organization) developed a vector control register in 2016, in accordance with the WHO Manual on Vector Surveillance and Control at Ports, Airports and Border Crossings [130]. Therefore, decisions are made on a global scale, but very few are implemented. More recently in 2019, a number of logistical, practical, and financial challenges in implementing additional disinsection requirements has been identified and recommendations made for further research, development, and implementation actions [130]. Accordingly, current efforts should lead to non-chemical insect control in order to overcome insecticide resistance and better prevent malaria in aircrafts, as well as in and around airports [130], along with early diagnosis and appropriate treatment of cases.

Currently, hopes have turned to biological diagnosis. Unfortunately, biologists in non-endemic areas are facing several shortcomings in performing and reading the thick drop, in detecting parasites on the smear of pauci-parasitic patients, and in determining the species when the parasite density is low [28,33]. These difficulties are sometimes increased by the previous use of drugs that alter the morphology of the parasites. In addition, the use of rapid diagnostic tests (RDT) poses problems in terms of sensitivity due to the deletion of the *pf*hrp 2/3 genes in *P. falciparum* [131,132,133]. Currently, more than 28 countries in Africa and southwest Asia have reported deletions of the *pf*hrp2/3 genes [1,134], which cause false negative RDT results in infection detection. In this situation, the probability of the patient to receive adequate treatment is low. This probability is even lower when it comes to airport malaria in non-endemic areas since most physicians have little reason to suspect it. In addition, the treatment initiated by the physician may be inappropriate due to the resistance of *Plasmodium* spp. to certain antimalarial drugs [28,88,135]. In the Southeast Asia region, failure rates for artemisinin-based combinations is above 10% [1]. In the Eastern Mediterranean region, the failure of sulfadoxine-pyrimethamine treatments against *P. falciparum* has led countries such as Somalia and Sudan to recommend artemether-lumefantrine as a first-line treatment [1]. Chloroquine is effective against *P. vivax* in most countries except Myanmar and East Timor [1]. For a better monitoring of the resistance of *Plasmodium* spp. to antimalarial drugs, it would be interesting to set up a resistance-mapping tool. The mapping tool will guide physicians in countries where malaria is not endemic to get an idea of the resistance of *Plasmodium* spp. to antimalarial drugs on a global scale. Nevertheless, the deployment of any intervention must be accompanied by rigorous routine entomological and epidemiological surveillance activities to monitor the success of the intervention and continuously inform the policies.

## 5. Conclusions

This review provides an update on the status of airport malaria in non-endemic areas. Although significant progress has been made in previous decades to reduce the disease burden worldwide, airport malaria is re-emerging in most countries of the European Union. This increase may be associated with climate change, growth of international travels, failure in aircraft disinsection, on top of delays in case diagnosis and treatment. Key issues hindering malaria case management and vector control interventions in and around the airports, e.g., delay in diagnosis and treatment, drug and insecticide resistance, technical and logistic shortcomings, should further be addressed by policy makers and disease control programmes. Moreover, the deployment of any intervention must be accompanied by rigorous routine entomological and epidemiological surveillance activities to inform policy in real time and monitor the effectiveness of the interventions.

## Figures and Tables

**Table 1:** Articles included in the review.

Articles Included (*n* = 27)	Thematic	References
11/27	Vectors bionomics, incrimination, and importation from endemic areas	[6,9,15,16,17,18,19,20,21,22,23,24]
7/27	Airport malaria epidemiological profiles	[24,25,26,27,28,29,30]
5/27	Diagnosis of *Plasmodium* spp. infections	[31,32,33,34,35]
3/27	Case management and treatment	[36,37,38]
7/27	Airport malaria mass prevention strategies	[9,22,23,24,25,26,28,39,40]

**Table 2:** Main antimalarial drugs for the treatment of malaria in adults [35,104].

Antimalarial	Treatment Line	Advantages	Disadvantages
Artenimol + Piperaquine	1st line	- Rapid parasitic clearance - Short treatment - Simple dosage - Taken on an empty stomach - General tolerance	- Conduction disorder - Drug interactions (CYP_3_A_4_ inhibitor)
Artemether + Lumefantrine	1st line	- Rapid parasitic clearance - Short treatment - General tolerance	- Low bioavailability (Lumefantrine) - Conduction disorders - Drug interaction (CYP_3_A_4_ inhibitor)
Atovaquone + Proguanil	2nd line	- Short treatment - General tolerance - Generics	- Vomiting - Low bioavailability
Quinine	3rd line	- Possible if pregnancy	- Medium tolerance - Long treatment

CYP_3_A_4:_ Cytochrome P450 3A4.

**Table 3:** Antimalarial drugs for the treatment of childhood malaria [35].

Antimalarial	Treatment Line	Advantages	Disadvantages
Artemether + Lumefantrine	1st line	- Rapid parasitic clearance - General tolerance	- QT interval prolongation without clinical translation - No suitable formulation for infants and small children - Prolonged duration of treatment
Artenimol + Piperaquine	1st line	- Rapid parasitic clearance - General tolerance - Taken on an empty stomach	- QT interval prolongation without clinical translation - No suitable formulation for infants and small children
Atovaquone + proguanil	2nd line	Cardiac tolerance	- No suitable dosage form for infants and small children - Prolonged duration of treatment - Digestive intolerance
Mefloquine	2nd line	- One socket per day - Cardiac tolerance	- No suitable dosage form for infants and small children - Digestive intolerance
Quinine	3rd line	Decline in use	- Cinchonism - Risk of intoxication - Prolonged duration of treatment

## Data Availability

The datasets supporting the findings of this article are included within the article.
